# The mechanism of ITGB4 in tumor migration and invasion

**DOI:** 10.3389/fonc.2024.1421902

**Published:** 2024-08-07

**Authors:** Guichen Huang, Minfeng Zhou, Damin Lu, Jinxiao Li, Qian Tang, Chutong Xiong, Fengxia Liang, Rui Chen

**Affiliations:** ^1^ Union Hospital Affiliated to Tongji Medical College, Huazhong University of Science and Technology, Wuhan, China; ^2^ School of Acupuncture and Bone Injury, Hubei University of Chinese Medicine, Wuhan, China

**Keywords:** integrin β4 (ITGb4), tumor metastasis, cell adhesion, epithelialmesenchymal transition (EMT), cancer therapy

## Abstract

Integrin β4 (ITGB4) is a transmembrane protein that functions as a mechanosensor, mediating the bidirectional exchange of information between the intracellular and extracellular matrices. ITGB4 plays a critical role in cell adhesion, migration, and signaling. Numerous studies have implicated ITGB4 as a key facilitator of tumor migration and invasion. This review provides a foundational description of the mechanisms by which ITGB4 regulates tumor migration and invasion through pathways involving focal adhesion kinase (FAK), protein kinase B (AKT), and matrix metalloproteinases (MMPs). These mechanisms encompass epithelial-mesenchymal transition (EMT), phosphorylation, and methylation of associated molecules. Additionally, this review explores the role of ITGB4 in the migration and invasion of prevalent clinical tumors, including those of the digestive system, breast, and prostate.

## 1 Introduction

Cancer poses a significant social, public health, and economic burden. Data from the International Agency for Research on Cancer (IARC) indicate that roughly one in five individuals, both men and women, will develop cancer during their lifetime. Furthermore, approximately one in nine men and one in two women succumb to the disease (1). Metastasis, the spread of cancer cells from the primary tumor to distant organs, is the leading cause of death from cancer. Unlike primary tumors, metastasis represents a systemic disease affecting the entire body (2). Therefore, elucidating the mechanisms underlying tumor metastasis and identifying non-specific targets within the metastatic cascade are crucial for advancing cancer therapy.

Integrins are heterodimeric transmembrane receptors composed of non-covalently associated α and β subunits. The human genome encodes 18 α and 8 β subunits, which can combine to form a repertoire of 24 distinct integrin receptors. Each integrin receptor possesses unique binding specificities and exhibits a distinct tissue distribution (3). These receptors span the plasma membrane and serve as platforms for the assembly of signaling complexes that physically connect the extracellular matrix to the intracellular cytoskeleton. Notably, integrins exhibit a remarkable capacity for bidirectional signaling across the plasma membrane, mediating both “inside-out” (4) and “outside-in” signaling (5). This bidirectional communication enables human cells to not only respond to changes in their surrounding extracellular environment but also actively influence it (6). It is now understood that integrins primarily mediate cell-matrix interactions and play crucial roles in various cellular processes, including adhesion, proliferation, differentiation, migration, and invasion (7).

Integrin β4 (ITGB4, also known as CD104), a member of the tryptophan-aspartic acid (WD)-40 repeat family, is a tumor-associated antigen (TAA) exhibiting high expression levels in diverse malignant tumors. ITGB4 promotes tumor progression by amplifying distinct signaling pathways (8) and facilitating tumor cell migration and invasion (9, 10). Transcriptome analysis from The Cancer Genome Atlas (TCGA) pinpointed *ITGB4* as a target gene in colorectal cancer, suggesting a critical role in disease development (11). Likewise, in squamous cell carcinoma of the lung, elevated ITGB4 expression is associated with tumorigenesis and progression via modulation of various signaling pathways (12). ITGB4 is widely recognized as a molecule with both prognostic and predictive value in cancer patients (13). Notably, ITGB4 exhibits high expression levels in oral squamous cell carcinoma, gliomas, and pancreatic cancer (14–17). In colon cancer patients, high ITGB4 expression correlates with poor overall survival. Additionally, *ITGB4* serves as a pivotal gene for constructing a predictive risk model for the clinical prognosis of lung adenocarcinoma (18–20). Furthermore, ITGB4 possesses diagnostic value for lung adenocarcinoma, demonstrating a significant correlation with overall survival (21).

ITGB4 has been implicated in promoting the invasive potential of various squamous cell carcinomas, breast cancers, and gastric cancers by activating MAPK and NF-κB signaling pathways (22–25). Mechanistically, ITGB4 engagement with extracellular ligands, such as human leukocyte antigen-1 (HLA-1), triggers the phosphorylation of Src kinase. This phosphorylation event subsequently leads to the activation of focal adhesion kinase (FAK) and downstream PI3K/AKT and Erk signaling pathways, ultimately promoting tumor cell migration (26). Furthermore, epidermal growth factor receptor (EGFR)/Src signaling can mediate the tyrosine phosphorylation of ITGB4, facilitating the recruitment of FAK to ITGB4. FAK activation then stimulates the Akt signaling pathway, thereby promoting tumor cell invasion (27–29). Collectively, these findings highlight the strong correlation between ITGB4 expression and tumor cell migration and invasion.

In contrast to other integrin β subunits, ITGB4 exclusively partners with the α6 subunit to form the α6β4 integrin, a cell adhesion molecule. Notably, within various cancers, α6β4 integrin undergoes release from hemidesmosomes. In this unbound state, α6β4 cooperates with growth factor receptors such as EGFR, ErbB-2, and c-Met. This cooperation leads to the amplification of downstream signaling pathways, including PI3K, AKT, MAPK, and Rho family GTPases. These activated pathways ultimately contribute to tumor migration and invasion (30). This study focused on the interplay between ITGB4 and other messenger RNAs (mRNAs) and molecules during its expression. We aimed to elucidate how ITGB4 interacts with these factors to influence tumor migration and invasion. By deciphering these mechanisms, we sought to provide valuable insights for further research exploring the impact of ITGB4 on cancer metastasis.

## 2 The interaction between ITGB4, FAK and AKT

Focal adhesion kinase is a central cytoplasmic protein tyrosine kinase. Functioning as a signaling hub, FAK integrates signals from various pathways and plays a critical role in promoting tumor cell invasion and migration through both kinase-dependent and -independent mechanisms. ITGB4 acts as an upstream regulator of FAK, influencing its expression and phosphorylation. For example, in ovarian cancer cells, Spectrin beta non-erythrocytic 2 (SPTBN2) may promote tumor migration and invasion by inhibiting the expression of focal adhesion-related proteins and downstream signaling pathways via ITGB4. Notably, overexpression of *ITGB4* reversed the decrease in phosphorylated FAK (p-FAK) induced by *SPTBN2* knockdown, while the combination of SPTBN2 knockdown and ITGB4 overexpression had no effect on total FAK protein expression (31). Furthermore, ITGB4 appears to influence FAK phosphorylation in various cancers. Spindle pole component 25 homologue (SPC25) preferentially affects genes associated with ECM-integrin interactions. Upregulation of *ITGB4* partially reverses the decrease in hepatocellular carcinoma cell invasion and migration caused by SPC25 silencing. Interestingly, both *SPC25* and *ITGB4* knockdown result in reduced phosphorylation of FAK, Phosphoinositide 3-kinase (PI3K), and AKT, all of which play crucial roles in these processes. In lung cancer, KCNF1, a regulator of epithelial-mesenchymal transition (EMT) and ECM-integrin interactions, positively regulates signaling downstream of ITGB4. Notably, the expression level of ITGB4 affects the phosphorylation of both FAK and AKT (32, 33).

The ITGB4/FAK signaling pathway is critical in regulating a diverse array of downstream molecules and signaling cascades associated with cancer cell metastasis. FAK activation, characterized by phosphorylation, is triggered upon cell adhesion to the extracellular matrix. This activation, in turn, initiates downstream signaling pathways intricately linked to cancer cell metastasis. Notably, the ITGB4/FAK pathway not only influences AKT phosphorylation but also modulates the activity of the FAK/AKT and PI3K/AKT signaling pathways. AKT, a central node within the PI3K/AKT pathway, exerts a profound influence on tumor cell proliferation, metastasis, invasion, and ultimately, patient prognosis (34).

ITGB4 functions as an upstream regulator of FAK, exerting a significant impact on AKT phosphorylation in non-small cell lung cancer (NSCLC) and hepatocellular carcinoma (32, 33). Studies further suggest that ITGB4 can activate the Akt signaling pathway by triggering FAK. These downstream effects of ITGB4 appear to be mediated by factors such as ZNF306 (35).

The interplay between ITGB4 and FAK can culminate in the inactivation of Akt and p38MAPK signaling pathways. Ziyuglycoside II, the primary compound isolated from *Sanguisorba officinalis L*., has been shown to inhibit the migration and invasion of triple-negative breast cancer (TNBC) cells. This inhibitory effect was mediated through Src/EGFR-dependent inactivation of the ITGB4/FAK signaling pathway, consequently leading to the inactivation of AKT and p38MAPK signaling pathways (36). EGFR has been found to interact with ITGB4 and influence anoikis through the ITGB4/FAK axis. By modulating the AKT pathway, this interaction plays a role in regulating cancer metastasis (28, 37, 38). Moreover, binding of PD-L1 to ITGB4 activates the AKT/GSK3β signaling pathway, thereby impacting the expression of the transcription inhibitor SNAIL and ultimately suppressing anti-tumor immunity (39).

EMT is a well-characterized cellular process during which epithelial cells undergo a phenotypic transformation, losing their epithelial characteristics and acquiring mesenchymal cell properties. This transition alters the adhesion molecules expressed on the cell surface, enabling cells to adopt migratory and invasive behaviors. EMT is demonstrably linked to tumor cell migration. A hallmark of EMT is the downregulation of the epithelial marker E-cadherin and the concomitant upregulation of the mesenchymal marker vimentin (40, 41). Notably, overexpression of ITGB4 has been shown to induce EMT by upregulating the transcription factor Slug. This upregulation leads to a loss of E-cadherin expression and the acquisition of a mesenchymal phenotype characterized by enhanced migratory and invasive capabilities (42). Conversely, the knockdown of ITGB4 in clear cell renal cell carcinoma (ccRCC) cells results in decreased expression of N-cadherin, vimentin, and the EMT-related transcription factor ZEB1 while simultaneously increasing E-cadherin expression (43).

Reciprocally, AKT can also function as an upstream regulator of ITGB4 expression, as evidenced by studies on NSCLC cells harboring pp53-R273H mutations. In these cells, AKT activation leads to a downregulation of ITGB4, thereby impacting their migratory and invasive phenotypes (44). This intricate interplay between ITGB4 and AKT highlights their critical roles in tumor cell migration and invasion. This interplay is further influenced by epidermal growth factor (EGF), various transcription factors, and drug monomers.

## 3 ITGB4 regulates the expression of MMPs

Matrix metalloproteinases (MMPs) are a family of proteases that play a critical role in shaping the extracellular matrix (ECM). Their expression is tightly regulated at the transcriptional level, and they are implicated in various cellular processes, including cell migration, invasion, angiogenesis, apoptosis, and inflammation (45). MMPs play a pivotal role in tumor progression, particularly in tumor migration and invasion, by facilitating the breakdown of histological barriers that normally restrict tumor cell movement. Studies have shown that nerve growth factor (NGF) promotes corneal epithelial migration by inducing the expression of MMP-9 (46). Furthermore, elevated expression of both MMP15 and ITGB4 has been observed in colorectal cancer patients following radical surgery. This finding suggests that ITGB4 may be involved in tumor progression by regulating MMPs (47). Supporting this notion, studies in ovarian cancer cells have demonstrated a positive correlation between the expression levels of ITGB4 and MMP2, MMP7, and MMP9 (31).

The mechanisms by which ITGB4 interacts with FAK, AKT and MMP to promote tumor migration and invasion are described in **Table 1**

**Table 1:** ITGB4 works with different molecules to regulate tumor migration and invasion.

Molecule	Model	Function of ITGB4	Machine	Phenotype	Reference
FAK/AKT	SPTBN2-knockdown A2780 cells	Reverse SPTBN2 knockout	Reverse decline in p-FAK expression	Reverse the decrease of p-FAK expression level	(31)
MMPs	A549, H23 and H2122 cells nude mouse Hepatocellular carcinoma cells and tissues ccRCC tissue and cells HCC tissue and cells MDA-MB-231 cells HCC tissue and cells ESCC tissue and cells - HCC tissue and cells ovarian cance cells H1299 cells HEK 293T cells Ovarian cells	Downstream of KCNF1 Downstream of SPC25 Enhancing the EMT process and facilitating metastasis ZKSCAN3 binds directly to the ITGB4 promoter - Increased susceptibility to anoikis ITGB4/FAK/Grb2 pathway ITGB4 directly bind PD-L1 - p53^R248^ induced cell adhesion NEU1 silencing increases ITGB4 protein and mRNA expression Focal adhesion and ECM receptor signaling pathways mediated by SPTBN2/IT GB4	Decreased phosphorylation of FAK Decreased phosphorylation of FAK/PI3K/AKT N6-methylamine modification of ITGB4 mRNA Triggers FAK to activate the AKT signaling pathway Src/EGFR effect ITGB4/FAK regulates Akt and p38MAPK pathways ITGB4-EGFR triggers FAK to activate the AKT signaling pathway phosphorylation of FAK and AKPde Activate the AKT/GSK3β signaling pathway Regulates SLug expression and AKT/Sox2-Nanog Activate the PI3K/Akt Pathway p53-R273H activates AKT signaling to promote NEU1 transcription The decline in MMP2, MMP7 and MMP9 levels was reversed	Reduce xenotransplantation in mice Promotes hepatocellular carcinoma metastasis Inhibit the metastasis of clear cell renal cell carcinoma Promotes hepatocellular carcinoma metastasis Inhibition of aggressive phenotype of triple-negative breast cancer cells Promotes lung metastasis of hepatocellular carcinoma Regulation of esophageal squamous cell carcinoma metastasis Promotes lymph node metastasis of cervical cancer Promotes hepatocellular cancer cell invasion and EMT Promote the adhesion of mesothelial cells Promote cancer cell migration Regulates the proliferation, invasion and migration of endometrial ovarian cancer cells	(32) (33) (43) (35) (36) (28) (38) (39) (48) (42) (44) (31)

## 4 The effect of ITGB4 on the migration and invasion of different tumors

The influence of ITGB4 on tumor migration and invasion extends beyond canonical signaling pathways. For instance, ITGB4 can interact with enzymes like 12-lipoxygenase (12-LOX), impacting tumor metastasis. Stimulation of ITGB4 triggers the recruitment of 12-LOX to the cell membrane, leading to its activation and subsequent production of 12(S)-HETE. This metabolite, in turn, regulates both angiogenesis and cell migration (49).

## 5 ITGB4 regulates the migration and invasion of digestive system related tumors

The multifaceted role of ITGB4 in tumor migration and invasion is further highlighted by its diverse effects across different cancer types. In esophageal cancer, the splice variant ITGB4E exhibits an inhibitory effect on esophageal squamous cell migration, while other variants appear to promote cell migration (50). Similarly, in gastric cancer, MPS-1 plays a regulatory role in invasion and migration by influencing ITGB4 expression (51). In colorectal cancer (CRC), Transcobalamin 1 (TCN1) disrupts the cytoskeletal network through modulation of ITGB4 signaling. Notably, *TCN1* knockdown exhibits a synergistic effect with ITGB4-mediated inactivation of Ki-67 and PCNA, further promoting CRC progression (52). Another study identified the transcriptional inhibitor CBX8 as a repressor of the ITGB4 promoter. Knockdown of *CBX8* leads to derepression and increased ITGB4 protein expression. This, in turn, reduces active RhoA, leading to actin rearrangements and enhanced CRC metastasis (53). Additionally, the microRNA miR-21 has been shown to regulate the colorectal cancer invasion-metastasis cascade by targeting ITGB4 (54). The influence of ITGB4 extends to hepatocellular carcinoma as well. In this context, ITGB4 overexpression downregulates the epithelial markers E-cadherin and N-cadherin, while simultaneously upregulating vimentin, p-AKT, Slug, Sox2, and Nanog in Bel-7402 or SMMC-77721 cells. These changes collectively induce epithelial-to-mesenchymal transition (EMT), promoting hepatocellular carcinoma invasion (48). Furthermore, ITGB4 functions as a laminin receptor, regulating bile duct cancer cell migration (55). In pancreatic ductal adenocarcinoma, ITGB4 promotes vimentin expression, induces EMT, and regulates migration and invasion (56).

Netrin-1, a guidance cue molecule, exhibits anti-tumorigenic properties in pancreatic ductal adenocarcinoma. Overexpression of netrin-1 has been shown to impede the growth of pancreatic ductal adenocarcinoma cells by downregulating *ITGB4* expression. Mechanistically, netrin-1 signaling through the UNC5B/FAK axis stimulates nitric oxide production. This, in turn, promotes the PP2A-mediated inhibition of the MEK/ERK pathway, ultimately leading to a reduction in the recruitment of phosphorylated c-Jun to ITGB4 promoters (57). The MEK/ERK pathway is a well-established signaling cascade implicated in various cellular processes, including gene expression, cell proliferation, and behaviors that influence tumor progression (58, 59). Activation of MEK rapidly triggers the phosphorylation of downstream ERK 1/2. These phosphorylated ERKs then activate transcription factors, kinases, and other signaling molecules, ultimately influencing tumor migration and invasion. Interestingly, studies have shown that phosphorylated ITGB4 at the Y1510 site can regulate the MEK1-ERK1/2 signaling cascade. Therefore, modulation of ITGB4 expression or its phosphorylation at Y1510 represents a potential novel therapeutic approach for pancreatic cancer (60). Furthermore, the MEK/ERK pathway can also regulate integrin α6β4 expression. KRAS mutations, frequently observed in pancreatic cancer, have been shown to regulate the expression of integrin α6β4 through the MEK/ERK pathway, thereby altering the migratory and invasive potential of tumor cells (61). Recepteur d’origine nantais (RON) receptor tyrosine kinases have been implicated in promoting the aggressive behavior of pancreatic cancer cells. These kinases disrupt the interaction between plectin and ITGB4, thereby stimulating cell migration. This disruption of the plectin-ITGB4 interaction is dependent on PI3K activity and RON signaling (62).

In summary, the multifaceted influence of ITGB4 on a diverse array of downstream signaling molecules underscores its critical role in promoting tumor migration and invasion across a spectrum of cancers.

The role of ITGB4 in digestive system tumor migration and invasion are described in **Table 2**.

**Table 2:** ITGB4 regulates the migration and invasion of digestive system related tumors.

Molecule	Model	Regulation of ITGB4	Machine	Phenotype	Reference
ITGB4E	OE21 cells	–	–	Slow down esophageal squamous cell migration	(50)
MPS-1	gastric adenocarcinoma cells	Negatively correlated with MPS-1	Changes in RNA and protein expression of ITGB4	Regulates the invasion and migration of gastric cancer cells	(51)
CBX8	CRC tissue and cells	Negatively correlated with CBX8	increase expression of the ITGB4 protein	Facilitates CRC migration, intrusion, and migration	(53)
miR-21 Slug Laminin - Netrin-1 RON	CRC cells HCC tissue and cells Cholangiocarcinoma cells Pancreatic cancer tissue CFPAC-1 cells AsPC-1 cells PDAC tissue and paracancerous tissue BxPC-3 cells(HEK)293 cells	Target gene of miR-21;Regulating EMT - As downstream signaling molecules for laminin Up regulating and promoting EMT The target of netrin-1 Combines with lectins to form hemasome	Regulate ITGB4 expression Regulates SLug expression and AKT/Sox2-Nanog in hepatocellular carcinoma Affected distribution of ITGB4 - UNC5B/FAK stimulate nitric oxide production, to promotes PP2A-mediated inhibition of the MEK/ERK and reduces recruitment of ITGB4 promoters by phosphorylated c-Jun RON binds to lectins and ITGB4, leading to disruption of lectin- ITGB4 interactions	miR-21 is overexpressed lines with high metastasis potential and EMT characteristics Promotes hepatocellular cancer cell invasion and EMT Induced migration process High expression of ITGB4 Inhibit the growth of xenografted PDAC cells Regulates the migration of pancreatic cancer cells	(54) (48) (55) (56) (57) (62)

## 6 ITGB4 regulates the migration and invasion of breast cancer

Caveolin-1 (P132L), a frequently occurring mutation in breast cancer, has been shown to selectively enhance the expression of ITGB4. ITGB4 is a well-characterized signaling molecule linked to tumor cell migration and invasion (63). Notably, overexpression of ITGB4 in triple-negative breast cancer cells has been implicated in the transfer of ITGB4 protein to cancer-associated fibroblasts (CAFs) via exosomes. This transfer, in turn, induces BNIP3L-dependent mitochondrial dysfunction and lactate production in CAFs. Co-culture assays revealed that the level of ITGB4 expression directly correlated with enhanced breast cancer cell proliferation, EMT, and invasion. Conversely, the knockdown of *ITGB4* or inhibition of exosome production in MDA-MB-231 cells, or blockade of c-Jun or AMPK phosphorylation in CAFs, significantly suppressed ITGB4-mediated mitochondrial autophagy and glycolysis in CAFs (9). Similar to ITGB4, Rac1 functions as a key regulator of the cytoskeleton. Both proteins represent promising therapeutic targets for disrupting the ability of cancer cells to reattach and establish themselves in new locations (reconnection ability or RA). Intriguingly, sustained Rac1 activity can prevent the lysosomal degradation of β4 integrins, highlighting a potential mechanism for its role in cancer progression (64).

SPARC acts as a downstream effector molecule that amplifies ITGB4-mediated invasion in breast cancer. However, the miR-29a microRNA directly targets SPARC, inhibiting this invasive process. Interestingly, breast cancer cells with low ITGB4 expression exhibit a concomitant decrease in miR-29a levels (65). TMEM268 is a transmembrane protein that interacts directly with the β4 subunit of integrin. Knockdown of TMEM268 promotes the ubiquitin-mediated degradation of ITGB4, ultimately leading to cytoskeletal remodeling (36).

The role of ITGB4 in breast cancer migration and invasion are described in **Table 3**.

**Table 3:** ITGB4 regulates the migration and invasion of breast cancer.

Molecule	Model	Function of ITGB4	Machine	Phenotype	Reference
Caveolin-1(P132L)	Met-1 cells WT mouse	–	Up regulated ITGB4 expression	Cell migration, invasion and metastasis increased significantly	(63)
–	–	Induce BNIP3L dependent mitochondrial and lactate production in CAFs	Overexpression of ITGB4 and provided to cancer-associated fibroblasts (CAF) through exosomes	Promote the proliferation, invasion and EMT of breast cancer cells	(9)
Rac1 SPARC Ziyuglycoside II	- breast cancer cells MDA-MB-231 cells	Affect reconnection ability Inhibition of miR-29a -	Rac1 activity regulates integrin levels in mammary epithelial cells Regulating SPARC Src/EGFR effect ITGB4/FAK regulates Akt and p38MAPK pathways	Regulates the ability of breast cancer cells to reattach Facilitated invasion Inhibition of aggressive phenotype of triple-negative breast cancer cells	(66) (67) (36)

## 7 ITGB4 regulates the migration and invasion of prostate cancer

ITGB4 has emerged as a critical driver of metastatic tumor cell migration and invasion in prostate cancer, as evidenced by studies using DU145 cells (68). ZEB1, a well-characterized regulator of EMT, interacts with the promoters of laminin α2 (LAMC2) and ITGB4, thereby influencing their expression levels (69). Interestingly, ITGB4 promoter methylation levels exhibit variation across prostate cancer cell lines representing distinct disease stages, including local tumors, lymph node metastases, and bone metastases (70). Estrogen receptor-regulated microRNA-182-5p (miR-182-5p) has been shown to promote proliferation, invasion, migration, and inhibit apoptosis in prostate cancer cells. This oncogenic effect is mediated through ARRDC3/ITGB4 signaling (71). Parathyroid hormone-related peptide (PTHrP) also contributes to prostate cancer growth and metastasis by regulating ITGB4 levels through transcriptional and post-translational pathways, including association with the NF-κB signaling pathway (72).

Proteomic analysis of exosomes isolated from body fluids has revealed upregulation of *ITGB4*. This finding suggests that ITGB4 may serve as a potential biomarker for prostate cancer progression and taxane resistance. Furthermore, the knockout of *ITGB4* in PC-3R cells significantly weakened their migratory and invasive capabilities, further highlighting the crucial role of ITGB4 in these processes (73).

The role of ITGB4 in prostate cancer migration and invasion are described in **Table 4**.

**Table 4:** ITGB4 regulates the migration and invasion of prostate cancer.

Molecule	Model	Function of ITGB4	Machine	Phenotype	Reference
–	DU145 cells nude mouse	Increased protein expression	Up regulated LAMC2 and ITGB4 mRNA and protein	The migration and invasion of metastatic DU145-LN cells increased	(68)
ZEB1	TEM4-18 cells	–	coordinately regulates laminin-332 and ITGB4 expression	Changes the invasive phenotype of prostate cancer cells	(69)
–	PC-3 cells LNCaP cells 22Rv1 cells	Differential methylation of different regions of the promoter	–	Different methylation regions represent different transfer modes	(70)
miR-182-5p PTHrP -	Prostate cancer tissue cells C4-2 cells PC-3 cells PC-3R cells	- Positively correlated with PTHrP -	miR-182-5p promotes PCa by affecting ITGB4 expression through ARRDC3 PTHrP regulates the expression level of ITGB4 ITGB4 and vinculin (VCL) in exosomes are upregulated	Promote the proliferation, invasion and migration of prostate cancer cells, and inhibit apoptosis Enhance CaP growth and metastasis *in vivo* Regulates migration and invasion of PC-3R cells	(71) (72) (73)

## 8 ITGB4 regulates the migration and invasion of other tumors

Quantitative real-time PCR analysis has identified a high ITGB4/JUP ratio as a significant factor promoting distant metastasis in oral cancer (74). This finding suggests that the relative expression level of ITGB4 compared to JUP may be a valuable prognostic indicator. Vimentin, an intermediate filament protein, has been shown to play a novel role in regulating cell motility by destabilizing adhesions mediated by β4 integrin. Interestingly, the knockdown of vimentin in oral cancer cells enhances the mechanical binding function of ITGB4 (75). This finding suggests a potential therapeutic strategy targeting the interaction between vimentin and ITGB4 to regulate cell motility and metastasis. Micropeptides that inhibit the actin cytoskeleton (MIACs) have been demonstrated to regulate interactions between the cytoskeletal protein SEPT2 and ITGB4. By suppressing the actin cytoskeleton, MIACs ultimately inhibit tumor growth and metastasis in head and neck squamous cell carcinoma (76).

In nasopharyngeal carcinoma, latent membrane protein 2A (LMP2A) competes with tyrosine kinase (ySyk) to bind ITGB4, promoting cell migration and invasion (77). Conversely, inhibition of LMP2A expression or miR-182-5p reduction leads to decreased migration and invasion, highlighting the potential regulatory role of these factors. Moreover, a meta-analysis of ccRCC identified ITGB4 as a target gene regulated by microRNA-204 (miR-204), further emphasizing the importance of ITGB4 in the context of cancer progression (78).

ITGB4 emerges as a key player with diverse regulatory mechanisms across different cancers. In brain cancer, mutations in the isocitrate dehydrogenase 1 (*IDH1*) gene, particularly IDH1^R132H/WT^, promote cell migration and inhibit proliferation by upregulating ITGB4, suggesting altered molecular targets and pathways (79). Ovarian cancer highlights another regulatory mechanism, where mutant *p53* promotes peritoneal metastasis by upregulating ITGB4 and activating the AKT pathway to enhance adhesion between cancer cells and mesothelial cells (42). Hypochlorous acid (HOCl) affects the oxidative modification of glucose-regulated protein 78 (GRP78) and GRP78 ATPase activity to regulate autophagy. ZBM-H acts as a probe for HOCl and can bind directly to GRP78 in the presence or absence of ATP. The interaction between GRP78 and annexin A7 (ANXA7) was facilitated after the use of ZBM-H, which was accompanied by an increased phosphorylation of ITGB4, which in turn regulated lung cancer cell behavior (80). In NSCLC, ZBM-H activates GRP78 ATPase, reduces ITGB4 protein levels, inhibits A549 cell migration, and suppresses EMT processes (81).

The role of ITGB4 in other tumors migration and invasion are described in **Table 5**

**Table 5:** ITGB4 regulates the migration and invasion of other tumors.

Molecule	Model	Function of ITGB4	Machine	Phenotype	Reference
–	Oral squamous cell carcinoma	A high ITGB4/JUP ratio was found to be a major factor in distant metastasis	–	Risk for local and blood-borne transmission of oral squamous cell carcinoma	(74)
vimentin	AW13516 cells	Linking vimentin intermediate filament proteins	Destabilize ITGB4 mediated binder interactions	Regulates cell adhesion	(75)
MIAC LMP2A - IDH1^R132H/WT^ p53^R248^ ZBM-H GRP78 ATPase	CAL27cells nude mouse - ccRCC and paracancerous tissue SVG-10B1cells U87 cells U373cells ovarian cancer cells A549 cells A549 cells	- LMP2A competes with tyrosine kinase to bind ITGB4 regulated by has-miR-204 Up regulation Participating in p53^R248^ induced cell adhesion ITGB4 phospho- rylation To regulate the selective autophagy and degradation of ITGB4	MIAC interacts directly with AQP2, regulating SEPT2/ITGB4 Syk interacts with ITGB4 - - Activate the PI3K/Akt pathway increased phosphorylation of ITGB4 Reduces ITGB4 protein levels in cells. Induce autophagy to negatively regulate ITGB4 protein levels	Inhibit tumor growth and metastasis to inhibit actin cytoskeleton Enhanced cell migration and invasion Correlated with focal adhesion of ccRCC Promote cell migration Promote the adhesion of ovarian cancer cells to mesothelial cells - Regulating cell migration	(76) (77) (78) (79) (42) (80) (81)

**Figure 1:**
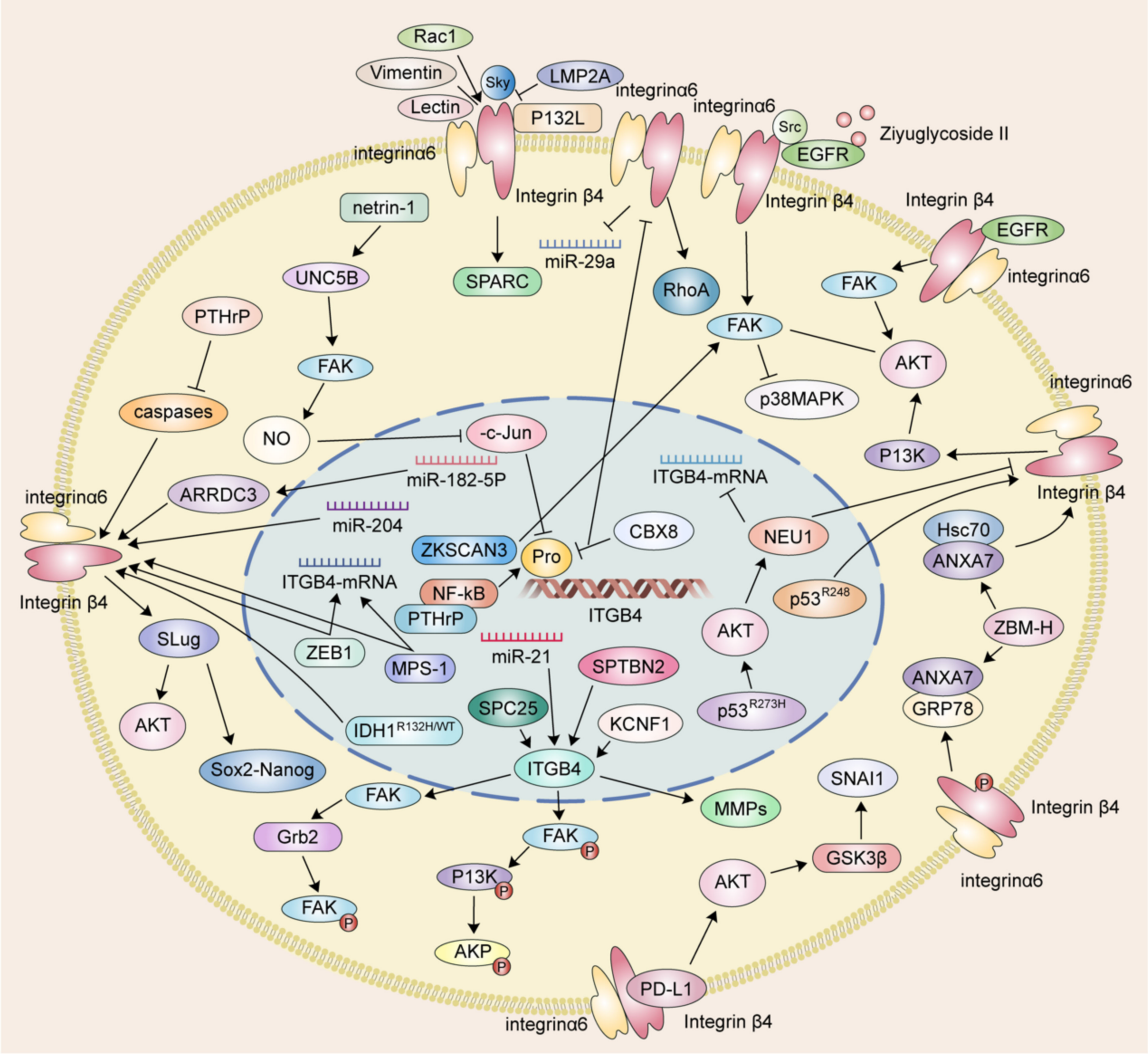
The mechanism of ITGB4 in tumor migration and invasion.

The mechanism of ITGB4 in tumor migration and invasion are described in **Figure 1**.

## 9 Conclusions

ITGB4 exhibits high expression levels in numerous tumors, making it a potential tumor marker. Furthermore, methylation and genetic alterations within the *ITGB4* gene hold significant promise for tumor diagnosis and treatment development (19, 82). Notably, the function of ITGB4 varies across different tumor types. In hepatocellular carcinoma and prostate cancer, ITGB4 expression is associated with tumor-associated fibroblasts. Conversely, in breast and lung cancers, ITGB4 correlates with the level of immune cell infiltration, suggesting potential roles in NA metabolism and protein processing within its functional mechanism (83). In gliomas, ITGB4 demonstrates high expression and serves as a reliable prognostic indicator for low-grade tumors, as confirmed by both bioinformatic analysis and tissue sample comparison (84). Integrins play a critical role in regulating various hallmarks of cancer, including tumor metastasis, immune evasion, and metabolic reprogramming. This has led to the exploration of integrin-targeted immunotherapy and other integrin inhibitors in preclinical and clinical studies (85). Interestingly, recent research suggests that ITGB4 may serve as a targeted immune site for CSCs (86). Furthermore, studies have revealed a novel signaling pathway in TNBC. TNFAIP2 promotes the proliferation and migration of TNBC cells by activating RAC1. ITGB4, through TNFAIP2 and IQGAP1, further activates RAC1, conferring resistance to DNA damage-induced cell death in TNBC. This ITGB4/TNFAIP2/IQGAP1/RAC1 signaling axis presents a potential therapeutic target to overcome DNA damage resistance in TNBC (87).

While research has confirmed the differential expression of upstream and downstream molecular pathways associated with ITGB4, a significant knowledge gap remains regarding its role in tumor biology, particularly its function as a cell adhesion molecule. Further studies are warranted to elucidate the precise molecular mechanisms by which ITGB4, a critical molecule in tumor progression and a potential target for therapeutic intervention, contributes to tumor development and metastasis.

## Author contributions

GH: Conceptualization, Writing – original draft, Writing – review & editing. MZ: Conceptualization, Writing – original draft, Writing – review & editing. DL: Conceptualization, Writing – original draft, Writing – review & editing. JL: Data curation, Formal analysis, Writing – original draft. QT: Data curation, Formal analysis, Writing – original draft. CX: Data curation, Formal analysis, Writing – original draft. FL: Supervision, Writing – original draft, Writing – review & editing. RC: Supervision, Writing – original draft, Writing – review & editing.

## References

[1] Bray F Laversanne M Sung H Ferlay J Siegel RL Soerjomataram I. Global cancer statistics 2022: GLOBOCAN estimates of incidence and mortality worldwide for 36 cancers in 185 countries. CA Cancer J Clin. (2024) 74:229–63. doi: 10.3322/caac.21834 38572751

[2] Ganesh K Massagué J. Targeting metastatic cancer. Nat Med. (2021) 27:34–44. doi: 10.1038/s41591-020-01195-4 33442008 PMC7895475

[3] Hynes RO. Integrins: bidirectional, allosteric signaling machines. Cell. (2002) 110:673–87. doi: 10.1016/S0092-8674(02)00971-6 12297042

[4] Faull RJ Ginsberg MH. Inside-out signaling through integrins. J Am Soc Nephrol. (1996) 7:1091–7. doi: 10.1681/ASN.V781091 8866399

[5] Zhu J Carman CV Kim M Shimaoka M Springer TA Luo BH. Requirement of alpha and beta subunit transmembrane helix separation for integrin outside-in signaling. Blood. (2007) 110:2475–83. doi: 10.1182/blood-2007-03-080077 PMC1988953 17615290

[6] Slack RJ Macdonald SJF Roper JA Jenkins RG Hatley RJD. Emerging therapeutic opportunities for integrin inhibitors. Nat Rev Drug Discovery. (2022) 21:60–78. doi: 10.1038/s41573-021-00284-4 34535788 PMC8446727

[7] Anderson LR Owens TW Naylor MJ. Structural and mechanical functions of integrins. Biophys Rev. (2014) 6:203–13. doi: 10.1007/s12551-013-0124-0 PMC5418412 28510180

[8] Ali SR Jordan M Nagarajan P Amit M. Nerve density and neuronal biomarkers in cancer. Cancers (Basel). (2022) 14. doi: 10.3390/cancers14194817 PMC9561962 36230740

[9] Sung JS Kang CW Kang S Jang Y Chae YC Kim BG. ITGB4-mediated metabolic reprogramming of cancer-associated fibroblasts. Oncogene. (2020) 39:664–76. doi: 10.1038/s41388-019-1014-0 31534187

[10] Qi L Knifley T Chen M O'Connor KL. Integrin α6β4 requires plectin and vimentin for adhesion complex distribution and invasive growth. J Cell Sci. (2022) 135. doi: 10.1242/jcs.258471 PMC8917354 34897465

[11] Chen B Ma Y Bi J Wang W He A Su G. Regulation network of colorectal-cancer-specific enhancers in the progression of colorectal cancer. Int J Mol Sci. (2021) 22. doi: 10.3390/ijms22158337 PMC8348541 34361106

[12] Zhong F Lu HP Chen G Dang YW Li GS Chen XY. The clinical significance and potential molecular mechanism of integrin subunit beta 4 in laryngeal squamous cell carcinoma. Pathol Res Pract. (2020) 216:152785. doi: 10.1016/j.prp.2019.152785 31889588

[13] Zhang M Liu Y Kong D. Erratum to "Identifying biomolecules and constructing a prognostic risk prediction model for recurrence in osteosarcoma. J Bone Oncol. (2021) 29:100371. doi: 10.1016/j.jbo.2021.100371 33376666 PMC7758551

[14] Chen X Yuan Q Liu J Xia S Shi X Su Y. Comprehensive characterization of extracellular matrix-related genes in PAAD identified a novel prognostic panel related to clinical outcomes and immune microenvironment: A silico analysis with in *vivo* and vitro validation. Front Immunol. (2022) 13:985911. doi: 10.3389/fimmu.2022.985911 36311789 PMC9606578

[15] Ma SR Liu JF Jia R Deng WW Jia J. Identification of a favorable prognostic subgroup in oral squamous cell carcinoma: Characterization of ITGB4/PD-L1(high) with CD8/PD-1(high). Biomolecules. (2023) 13. doi: 10.3390/biom13061014 PMC10296360 37371594

[16] Ma B Zhang L Zou Y He R Wu Q Han C. Reciprocal regulation of integrin β4 and KLF4 promotes gliomagenesis through maintaining cancer stem cell traits. J Exp Clin Cancer Res. (2019) 38:23. doi: 10.1186/s13046-019-1034-1 30658712 PMC6339386

[17] Lévy P Vidaud D Leroy K Laurendeau I Wechsler J Bolasco G. Molecular profiling of Malignant peripheral nerve sheath tumors associated with neurofibromatosis type 1, based on large-scale real-time RT-PCR. Mol Cancer. (2004) 3:20. doi: 10.1186/1476-4598-3-20 15255999 PMC493279

[18] Guo W Zhao G Liu S Deng T Zhang G Zhang B. Development of the prognostic value in lung adenocarcinoma based on anoikis-related genes and initial experimental validation. J Gene Med. (2023) 25:e3534. doi: 10.1002/jgm.3534 37259225

[19] Huang W Fan L Tang Y Chi Y Li J. A pan-cancer analysis of the oncogenic role of integrin beta4 (ITGB4) in human tumors. Int J Gen Med. (2021) 14:9629–45. doi: 10.2147/IJGM.S341076 PMC8674675 34924769

[20] Li M Jiang X Wang G Zhai C Liu Y Li H. ITGB4 is a novel prognostic factor in colon cancer. J Cancer. (2019) 10:5223–33. doi: 10.7150/jca.29269 PMC6775604 31602273

[21] Guo Q Liu XL Liu HS Luo XY Yuan Y Ji YM. The risk model based on the three oxidative stress-related genes evaluates the prognosis of LAC patients. Oxid Med Cell Longev. (2022) 2022:4022896. doi: 10.1155/2022/4022896 35783192 PMC9246616

[22] Shaw LM Rabinovitz I Wang HH Toker A Mercurio AM. Activation of phosphoinositide 3-OH kinase by the alpha6beta4 integrin promotes carcinoma invasion. Cell. (1997) 91:949–60. doi: 10.1016/S0092-8674(00)80486-9 9428518

[23] Lipscomb EA Mercurio AM. Mobilization and activation of a signaling competent alpha6beta4integrin underlies its contribution to carcinoma progression. Cancer Metastasis Rev. (2005) 24:413–23. doi: 10.1007/s10555-005-5133-4 16258729

[24] Nikolopoulos SN Blaikie P Yoshioka T Guo W Puri C Tacchetti C. Targeted deletion of the integrin beta4 signaling domain suppresses laminin-5-dependent nuclear entry of mitogen-activated protein kinases and NF-kappaB, causing defects in epidermal growth and migration. Mol Cell Biol. (2005) 25:6090–102. doi: 10.1128/MCB.25.14.6090-6102.2005 PMC1168825 15988021

[25] Yoon SO Shin S Lipscomb EA. A novel mechanism for integrin-mediated ras activation in breast carcinoma cells: the alpha6beta4 integrin regulates ErbB2 translation and transactivates epidermal growth factor receptor/ErbB2 signaling. Cancer Res. (2006) 66:2732–9. doi: 10.1158/0008-5472.CAN-05-2941 16510594

[26] Zhang X Rozengurt E Reed EF. HLA class I molecules partner with integrin β4 to stimulate endothelial cell proliferation and migration. Sci Signal. (2010) 3:ra85. doi: 10.1126/scisignal.2001158 21098729 PMC3878299

[27] Margadant C Frijns E Wilhelmsen K Sonnenberg A. Regulation of hemidesmosome disassembly by growth factor receptors. Curr Opin Cell Biol. (2008) 20:589–96. doi: 10.1016/j.ceb.2008.05.001 18583123

[28] Leng C Zhang ZG Chen WX Luo HP Song J Dong W. An integrin beta4-EGFR unit promotes hepatocellular carcinoma lung metastases by enhancing anchorage independence through activation of FAK-AKT pathway. Cancer Lett. (2016) 376:188–96. doi: 10.1016/j.canlet.2016.03.023 26996299

[29] Tai YL Chu PY Lai IR Wang MY Tseng HY Guan JL. An EGFR/Src-dependent β4 integrin/FAK complex contributes to Malignancy of breast cancer. Sci Rep. (2015) 5:16408. doi: 10.1038/srep16408 26549523 PMC4637903

[30] Stewart RL O'Connor KL. Clinical significance of the integrin α6β4 in human Malignancies. Lab Invest. (2015) 95:976–86. doi: 10.1038/labinvest.2015.82 PMC4554527 26121317

[31] Yang L Gu Y. SPTBN2 regulates endometroid ovarian cancer cell proliferation, invasion and migration *via* ITGB4−mediated focal adhesion and ECM receptor signalling pathway. Exp Ther Med. (2023) 25:277. doi: 10.3892/etm 37206547 PMC10189743

[32] Shi WK Shang QL Zhao YF. SPC25 promotes hepatocellular carcinoma metastasis via activating the FAK/PI3K/AKT signaling pathway through ITGB4. Oncol Rep. (2022) 47. doi: 10.3892/or PMC8968763 35293598

[33] Chen CY Wu PY Van Scoyk M Simko SA Chou CF Winn RA. KCNF1 promotes lung cancer by modulating ITGB4 expression. Cancer Gene Ther. (2023) 30:414–23. doi: 10.1038/s41417-022-00560-4 PMC10014577 36385523

[34] Song M Bode AM Dong Z Lee MH. AKT as a therapeutic target for cancer. Cancer Res. (2019) 79:1019–31. doi: 10.1158/0008-5472.CAN-18-2738 30808672

[35] Li J Hao N Han J Zhang M Li X Yang N. ZKSCAN3 drives tumor metastasis *via* integrin β4/FAK/AKT mediated epithelial-mesenchymal transition in hepatocellular carcinoma. Cancer Cell Int. (2020) 20:216. doi: 10.1186/s12935-020-01307-7 32518525 PMC7275473

[36] Wang K Zou P Zhu X Zhang T. Ziyuglycoside II suppresses the aggressive phenotype of triple negative breast cancer cells through regulating Src/EGFR-dependent ITGB4/FAK signaling. Toxicol In Vitro. (2019) 61:104653. doi: 10.1016/j.tiv.2019.104653 31525383

[37] Schinke H Shi E Lin Z Quadt T Kranz G Zhou J. A transcriptomic map of EGFR-induced epithelial-to-mesenchymal transition identifies prognostic and therapeutic targets for head and neck cancer. Mol Cancer. (2022) 21:178. doi: 10.1186/s12943-022-01646-1 36076232 PMC9454230

[38] Zhu JF Liu Y Huang H Shan L Han ZG Liu JY. MicroRNA-133b/EGFR axis regulates esophageal squamous cell carcinoma metastases by suppressing anoikis resistance and anchorage-independent growth. Cancer Cell Int. (2018) 18:193. doi: 10.1186/s12935-018-0684-y 30479571 PMC6251163

[39] Wang S Li J Xie J Liu F Duan Y Wu Y. Programmed death ligand 1 promotes lymph node metastasis and glucose metabolism in cervical cancer by activating integrin β4/SNAI1/SIRT3 signaling pathway. Oncogene. (2018) 37:4164–80. doi: 10.1038/s41388-018-0252-x 29706653

[40] Nieto MA Huang RY Jackson RA Thiery JP. EMT: 2016. Cell. (2016) 166:21–45. doi: 10.1016/j.cell.2016.06.028 27368099

[41] Puisieux A Brabletz T Caramel J. Oncogenic roles of EMT-inducing transcription factors. Nat Cell Biol. (2014) 16:488–94. doi: 10.1038/ncb2976 24875735

[42] Lee JG Ahn JH Jin Kim T Ho Lee J Choi JH. Mutant p53 promotes ovarian cancer cell adhesion to mesothelial cells *via* integrin β4 and Akt signals. Sci Rep. (2015) 5:12642. doi: 10.1038/srep12642 26223322 PMC4649895

[43] Liu Z Sun T Piao C Zhang Z Kong C. METTL14-mediated N(6)-methyladenosine modification of ITGB4 mRNA inhibits metastasis of clear cell renal cell carcinoma. Cell Commun Signal. (2022) 20:36. doi: 10.1186/s12964-022-00831-5 35305660 PMC8934459

[44] Lv T Lv H Fei J Xie Y Lian D Hu J. p53-R273H promotes cancer cell migration *via* upregulation of neuraminidase-1. J Cancer. (2020) 11:6874–82. doi: 10.7150/jca.44718 PMC7591995 33123278

[45] de Almeida LGN Thode H Eslambolchi Y Chopra S Young D Gill S. Matrix metalloproteinases: From molecular mechanisms to physiology, pathophysiology, and pharmacology. Pharmacol Rev. (2022) 74:712–68. doi: 10.1124/pharmrev.121.000349 35738680

[46] Blanco-Mezquita T Martinez-Garcia C Proença R Zieske JD Bonini S Lambiase A. Nerve growth factor promotes corneal epithelial migration by enhancing expression of matrix metalloprotease-9. Invest Ophthalmol Vis Sci. (2013) 54:3880–90. doi: 10.1167/iovs.12-10816 PMC5110072 23640040

[47] Ortega P Moran A Fernandez-Marcelo T De Juan C Frias C Lopez-Asenjo JA. MMP-7 and SGCE as distinctive molecular factors in sporadic colorectal cancers from the mutator phenotype pathway. Int J Oncol. (2010) 36:1209–15. doi: 10.3892/ijo_00000604 20372795

[48] Li XL Liu L Li DD He YP Guo LH Sun LP. Integrin β4 promotes cell invasion and epithelial-mesenchymal transition through the modulation of Slug expression in hepatocellular carcinoma. Sci Rep. (2017) 7:40464. doi: 10.1038/srep40464 28084395 PMC5233967

[49] Liu SY Ge D Chen LN Zhao J Su L Zhang SL. A small molecule induces integrin β4 nuclear translocation and apoptosis selectively in cancer cells with high expression of integrin β4. Oncotarget. (2016) 7:16282–96. doi: 10.18632/oncotarget.v7i13 PMC4941314 26918348

[50] Yang ZY Jiang H Qu Y Wei M Yan M Zhu ZG. Metallopanstimulin-1 regulates invasion and migration of gastric cancer cells partially through integrin β4. Carcinogenesis. (2013) 34:2851–60. doi: 10.1093/carcin/bgt226 23803695

[51] Kelly GT Faraj R Dai Z Cress AE Wang T. A mutation found in esophageal cancer alters integrin beta4 mRNA splicing. Biochem Biophys Res Commun. (2020) 529:726–32. doi: 10.1016/j.bbrc.2020.06.078 32736699

[52] Zhu X Jiang X Zhang Q Huang H Shi X Hou D. TCN1 deficiency inhibits the Malignancy of colorectal cancer cells by regulating the ITGB4 pathway. Gut Liver. (2023) 17:412–29. doi: 10.5009/gnl210494 PMC10191790 35686504

[53] Tang J Wang G Zhang M Li FY Sang Y Wang B. Paradoxical role of CBX8 in proliferation and metastasis of colorectal cancer. Oncotarget. (2014) 5:10778–90. doi: 10.18632/oncotarget.v5i21 PMC4279409 25360999

[54] Ferraro A Kontos CK Boni T Bantounas I Siakouli D Kosmidou V. Epigenetic regulation of miR-21 in colorectal cancer: ITGB4 as a novel miR-21 target and a three-gene network (miR-21-ITGΒ4-PDCD4) as predictor of metastatic tumor potential. Epigenetics. (2014) 9:129–41. doi: 10.4161/epi.26842 PMC3928175 24149370

[55] Islam K Thummarati P Kaewkong P Sripa B Suthiphongchai T. Role of laminin and cognate receptors in cholangiocarcinoma cell migration. Cell Adh Migr. (2021) 15:152–65. doi: 10.1080/19336918.2021.1924422 PMC8143218 34014802

[56] Masugi Y Yamazaki K Emoto K Effendi K Tsujikawa H Kitago M. Upregulation of integrin β4 promotes epithelial-mesenchymal transition and is a novel prognostic marker in pancreatic ductal adenocarcinoma. Lab Invest. (2015) 95:308–19. doi: 10.1038/labinvest.2014.166 25599535

[57] An XZ Zhao ZG Luo YX Zhang R Tang XQ Hao D. Netrin-1 suppresses the MEK/ERK pathway and ITGB4 in pancreatic cancer. Oncotarget. (2016) 7:24719–33. doi: 10.18632/oncotarget.v7i17 PMC5029736 27034160

[58] Wang C Li Z Shao F Yang X Feng X Shi S. High expression of Collagen Triple Helix Repeat Containing 1 (CTHRC1) facilitates progression of oesophageal squamous cell carcinoma through MAPK/MEK/ERK/FRA-1 activation. J Exp Clin Cancer Res. (2017) 36:84. doi: 10.1186/s13046-017-0555-8 28645305 PMC5481965

[59] Iezzi A Caiola E Scagliotti A Broggini M. Generation and characterization of MEK and ERK inhibitors- resistant non-small-cells-lung-cancer (NSCLC) cells. BMC Cancer. (2018) 18:1028. doi: 10.1186/s12885-018-4949-6 30352565 PMC6199806

[60] Meng X Liu P Wu Y Liu X Huang Y Yu B. Integrin beta 4 (ITGB4) and its tyrosine-1510 phosphorylation promote pancreatic tumorigenesis and regulate the MEK1-ERK1/2 signaling pathway. Bosn J Basic Med Sci. (2020) 20:106–16. doi: 10.17305/bjbms.2019.4255 PMC7029197 31242404

[61] Choi SH Kim JK Chen CT Wu C Marco MR Barriga FM. KRAS mutants upregulate integrin β4 to promote invasion and metastasis in colorectal cancer. Mol Cancer Res. (2022) 20:1305–19. doi: 10.1158/1541-7786.MCR-21-0994 PMC9357101 35394541

[62] Yu PT Babicky M Jaquish D French R Marayuma K Mose E. The RON-receptor regulates pancreatic cancer cell migration through phosphorylation-dependent breakdown of the hemidesmosome. Int J Cancer. (2012) 131:1744–54. doi: 10.1002/ijc.27447 PMC3424378 22275185

[63] Bonuccelli G Casimiro MC Sotgia F Wang C Liu M Katiyar S. Caveolin-1 (P132L), a common breast cancer mutation, confers mammary cell invasiveness and defines a novel stem cell/metastasis-associated gene signature. Am J Pathol. (2009) 174:1650–62. doi: 10.2353/ajpath.2009.080648 PMC2671254 19395651

[64] Mori K Higurashi M Ishikawa F Shibanuma M. Rac1-mediated sustained β4 integrin level develops reattachment ability of breast cancer cells after anchorage loss. Cancer Sci. (2021) 112:3205–17. doi: 10.1111/cas.14985 PMC8353950 34036687

[65] Gerson KD Shearstone JR Maddula V Seligmann BE Mercurio AM. Integrin β4 regulates SPARC protein to promote invasion. J Biol Chem. (2012) 287:9835–44. doi: 10.1074/jbc.M111.317727 PMC3323016 22308039

[66] Mori K Higurashi M Ishikawa F Shibanuma M. Rac1-mediated sustained beta4 integrin level develops reattachment ability of breast cancer cells after anchorage loss. Cancer Sci. (2021) 112:3205–17. doi: 10.1111/cas.14985 PMC8353950 34036687

[67] Gerson KD Shearstone JR Maddula V Seligmann BE Mercurio AM. Integrin beta4 regulates SPARC protein to promote invasion. J Biol Chem. (2012) 287:9835–44. doi: 10.1074/jbc.M111.317727 PMC3323016 22308039

[68] Banyard J Chung I Migliozzi M Phan DT Wilson AM Zetter BR. Identification of genes regulating migration and invasion using a new model of metastatic prostate cancer. BMC Cancer. (2014) 14:387. doi: 10.1186/1471-2407-14-387 24885350 PMC4046438

[69] Drake JM Barnes JM Madsen JM Domann FE Stipp CS Henry MD. ZEB1 coordinately regulates laminin-332 and {beta}4 integrin expression altering the invasive phenotype of prostate cancer cells. J Biol Chem. (2010) 285:33940–8. doi: 10.1074/jbc.M110.136044 PMC2962494 20729552

[70] Wilkinson EJ Woodworth AM Parker M Phillips JL Malley RC Dickinson JL. Epigenetic regulation of the ITGB4 gene in prostate cancer. Exp Cell Res. (2020) 392:112055. doi: 10.1016/j.yexcr.2020.112055 32376286

[71] Yao J Xu C Fang Z Li Y Liu H Wang Y. Androgen receptor regulated microRNA miR-182-5p promotes prostate cancer progression by targeting the ARRDC3/ITGB4 pathway. Biochem Biophys Res Commun. (2016) 474:213–9. doi: 10.1016/j.bbrc.2016.04.107 27109471

[72] Bhatia V Mula RV Falzon M. Parathyroid hormone-related protein regulates integrin α6 and β4 levels *via* transcriptional and post-translational pathways. Exp Cell Res. (2013) 319:1419–30. doi: 10.1016/j.yexcr.2013.03.003 PMC3773845 23499737

[73] Kawakami K Fujita Y Kato T Mizutani K Kameyama K Tsumoto H. Integrin β4 and vinculin contained in exosomes are potential markers for progression of prostate cancer associated with taxane-resistance. Int J Oncol. (2015) 47:384–90. doi: 10.3892/ijo.2015.3011 25997717

[74] Nagata M Noman AA Suzuki K Kurita H Ohnishi M Ohyama T. ITGA3 and ITGB4 expression biomarkers estimate the risks of locoregional and hematogenous dissemination of oral squamous cell carcinoma. BMC Cancer. (2013) 13:410. doi: 10.1186/1471-2407-13-410 24006899 PMC3844399

[75] Dmello C Sawant S Alam H Gangadaran P Tiwari R Dongre H. Vimentin-mediated regulation of cell motility through modulation of beta4 integrin protein levels in oral tumor derived cells. Int J Biochem Cell Biol. (2016) 70:161–72. doi: 10.1016/j.biocel.2015.11.015 26646105

[76] Li M Li X Zhang Y Wu H Zhou H Ding X. Micropeptide MIAC inhibits HNSCC progression by interacting with aquaporin 2. J Am Chem Soc. (2020) 142:6708–16. doi: 10.1021/jacs.0c00706 32176498

[77] Zhou X Matskova L Rathje LS Xiao X Gish G Werner M. SYK interaction with ITGβ4 suppressed by Epstein-Barr virus LMP2A modulates migration and invasion of nasopharyngeal carcinoma cells. Oncogene. (2015) 34:4491–9. doi: 10.1038/onc.2014.380 25531330

[78] Han Y Wang L Wang Y. Integrated analysis of three publicly available gene expression profiles identified genes and pathways associated with clear cell renal cell carcinoma. Med Sci Monit. (2020) 26:e919965. doi: 10.12659/MSM.919965 32712616 PMC7405617

[79] Wei S Wang J Oyinlade O Ma D Wang S Kratz L. Heterozygous IDH1(R132H/WT) created by "single base editing" inhibits human astroglial cell growth by downregulating YAP. Oncogene. (2018) 37:5160–74. doi: 10.1038/s41388-018-0334-9 PMC6590918 29849122

[80] Ning J Wang X Li N Cui X Li N Zhao B. ZBM-H-induced activation of GRP78 ATPase promotes apoptosis *via* annexin A7 in A549 lung cancer cells. J Cell Biochem. (2022) 123:798–806. doi: 10.1002/jcb.30224 35118704

[81] Ning J Cui X Li N Li N Zhao B Miao J. Activation of GRP78 ATPase suppresses A549 lung cancer cell migration by promoting ITGB4 degradation. Cell Adh Migr. (2022) 16:107–14. doi: 10.1080/19336918.2022.2130415 PMC9542429 36203272

[82] Yang H Xu Z Peng Y Wang J Xiang Y. Integrin β4 as a potential diagnostic and therapeutic tumor marker. Biomolecules. (2021) 11. doi: 10.3390/biom11081197 PMC8394641 34439865

[83] Carlow TJ. Medical treatment of nystagmus and ocular motor disorders. Int Ophthalmol Clin. (1986) 26:251–64. doi: 10.1097/00004397-198602640-00022 3492476

[84] Chen P Ma T Yan T Song Z Liu C Pan C. ITGB4 upregulation is associated with progression of lower grade glioma. Sci Rep. (2024) 14:421. doi: 10.1038/s41598-023-49801-y 38172503 PMC10764336

[85] Liu F Wu Q Dong Z Liu K. Integrins in cancer: Emerging mechanisms and therapeutic opportunities. Pharmacol Ther. (2023) 247:108458. doi: 10.1016/j.pharmthera.2023.108458 37245545

[86] Ruan S Lin M Zhu Y Lum L Thakur A Jin R. Integrin β4-targeted cancer immunotherapies inhibit tumor growth and decrease metastasis. Cancer Res. (2020) 80:771–83. doi: 10.1158/0008-5472.CAN-19-1145 PMC7024642 31843981

[87] Fang H Ren W Cui Q Liang H Yang C Liu W. Integrin β4 promotes DNA damage-related drug resistance in triple-negative breast cancer *via* TNFAIP2/IQGAP1/RAC1. Elife. (2023) 12. doi: 10.7554/eLife.88483 PMC10547475 37787041

